# Introduction to the shared near infrared spectroscopy format

**DOI:** 10.1117/1.NPh.10.1.013507

**Published:** 2022-12-09

**Authors:** Stephen Tucker, Jay Dubb, Sreekanth Kura, Alexander von Lühmann, Robert Franke, Jörn M. Horschig, Samuel Powell, Robert Oostenveld, Michael Lührs, Édouard Delaire, Zahra M. Aghajan, Hanseok Yun, Meryem A. Yücel, Qianqian Fang, Theodore J. Huppert, Blaise B. Frederick, Luca Pollonini, David Boas, Robert Luke

**Affiliations:** a Boston University, Neurophotonics Center, Department of Biomedical Engineering, Boston, Massachusetts, United States; b NIRx Medical Technologies, Berlin, Germany; c Artinis Medical Systems B.V., Elst, The Netherlands; d Gowerlabs, London, United Kingdom; e University of Nottingham, Nottingham, United Kingdom; f University College London, London, United Kingdom; g Radboud University, Donders Institute for Brain, Cognition and Behaviour, Nijmegen, The Netherlands; h Karolinska Institutet, NatMEG, Stockholm, Sweden; i Maastricht University, Maastricht, The Netherlands; j Brain Innovation B.V., Maastricht, The Netherlands; k Concordia University, Montreal, Quebec, Canada; l Kernel, Los Angeles, California, United States; m OBELAB, Seoul, Republic of Korea; n Northeastern University, Department of Bioengineering, Boston, Massachusetts, United States; o University of Pittsburgh, Pittsburgh, Pennsylvania, United States; p Mclean Hospital, Brain Imaging Center, Belmont, Massachusetts, United States; q Harvard Medical School, Department of Psychiatry, Boston, Massachusetts, United States; r University of Houston, Houston, Texas, United States; s Macquarie University, Sydney, Australia

**Keywords:** functional near-infrared spectroscopy, shared near-infrared spectroscopy format, standardization, data format, data sharing, software

## Abstract

**Significance:**

Functional near-infrared spectroscopy (fNIRS) is a popular neuroimaging technique with proliferating hardware platforms, analysis approaches, and software tools. There has not been a standardized file format for storing fNIRS data, which has hindered the sharing of data as well as the adoption and development of software tools.

**Aim:**

We endeavored to design a file format to facilitate the analysis and sharing of fNIRS data that is flexible enough to meet the community’s needs and sufficiently defined to be implemented consistently across various hardware and software platforms.

**Approach:**

The shared NIRS format (SNIRF) specification was developed in consultation with the academic and commercial fNIRS community and the Society for functional Near Infrared Spectroscopy.

**Results:**

The SNIRF specification defines a format for fNIRS data acquired using continuous wave, frequency domain, time domain, and diffuse correlation spectroscopy devices.

**Conclusions:**

We present the SNIRF along with validation software and example datasets. Support for reading and writing SNIRF data has been implemented by major hardware and software platforms, and the format has found widespread use in the fNIRS community.

## 1 Introduction

Functional near-infrared spectroscopy (fNIRS) is a family of noninvasive neuroimaging techniques that estimate concentrations of oxygenated and deoxygenated hemoglobin near the surface of the brain and thus infer relative changes in neural activation.^1^ There are various technical approaches to NIRS measurements, most prominently the continuous-wave, frequency domain, and time-domain techniques. Each approach provides unique benefits, utilizes different instrumentation, and yields different raw data.^1^^,^^2^

In recent years, fNIRS has increased in utility with its adoption across a range of scientific fields and with the development of portable devices, which has enabled experiments beyond traditional lab-based evoked response paradigms,^3^^,^^4^ for instance, the recording of multiple subjects simultaneously^5^ or the capture of rich environmental metadata. In parallel, probes have increased in size: an fNIRS study can be conducted using only a single “optode,” whereas the most advanced arrangements consist of many, generating large amounts of data to create sophisticated and informative maps of neural activation. The latest commercially produced systems are capable of yielding recordings from hundreds of channels at hundreds of samples per second.^6^

To capitalize on the increasing amount and variety of data, a range of software packages have emerged to democratize data analysis techniques, with each providing unique insights.^7^^–^^16^ However, without a clearly defined data storage standard, sharing data between these platforms has been difficult. Similarly, acquisition systems from various hardware vendors (including Hitachi, TechEn, Shimadzu, Gowerlabs, NIRx, Artinis Medical Systems, ISS, Rogue Research, OBELAB, and Kernel) have historically lacked a unified export format, burdening members of the fNIRS community with the engineering of platform specific Input/Output and conversion applications.

Ideally, data produced by acquisition systems would be immediately compatible with the various analysis and visualization software packages developed by both academic and commercial parties, easing collaboration and the design of experiments and processing pipelines that take advantage of the latest technologies. To date, vendors and researchers have organized their data using a plethora of methods, including text files or binary formats that are platform specific, such as MATLAB’s MAT-file (MathWorks, Natick, Massachusetts, United States). This has limited the ability of colleagues to share data and leverage the strengths of various software packages.

Increased access to shared data increases transparency and reproducibility^17^ and pushes the scientific field forward through new discoveries.^18^ At an individual level, the sharing of data accelerates the entrance of new researchers to the field. Standardized, open-access neuroimaging datasets enable researchers to focus on their primary objectives rather than learning the idiosyncratic details of specific fNIRS software and hardware.

To this end, we introduce the shared NIRS format (SNIRF). This format was designed to standardize digital representations of fNIRS data and to provide a complete set of its descriptors. The format specifies ubiquitous fNIRS data features but extends to mode-specific details, stimulus information, and metadata. As of 2022, the format has been implemented by a range of hardware and software platforms.

## 2 Development Process

SNIRF is the result of collaboration between a growing number of researchers and commercial representatives that began in 2014. During the early stage of the development, the considerations of academic and commercial parties were collected and formulated into a draft specification. The SNIRF specification was progressively designed in public via the GitHub website. This allowed users and developers to provide feedback and to propose changes to the specification in an open and transparent manner.

As the needs of the fNIRS community grow and new hardware and experimental processes are developed, we anticipate that extensions and refinements to the SNIRF file format will be implemented in future versions. Changes in the specification are identified using semantic versioning.^19^ This allows researchers and developers to guarantee compliance with the file format and increases interoperability. The SNIRF specification will continue to be improved, and we welcome feedback and contributions from the fNIRS community.

### 2.1 Governance

SNIRF’s governance structure is intended to ensure the format’s longevity by enabling the fNIRS community to expand or correct the specification without introducing volatile changes that cause problems for users. The specification can be modified by its maintainers via a GitHub repository: edits to the document can be proposed and triaged in response to discussions in the Issues forum. Releases of SNIRF are finalized by a small steering committee. Although anyone with a GitHub account can participate in discussions and propose changes to drafts of the specification document, only users with the title of maintainer can alter, approve, or merge these changes. Maintainers are appointed by the steering committee from the most active and experienced contributors to the specification.

The steering committee is composed of an odd-numbered group of senior stakeholders that are ultimately responsible for decision-making and guiding the project toward its goals. The steering committee must unanimously approve each release of the specification. Should a member wish to leave the committee, a replacement must be appointed and approved by a majority of the maintainers and current committee members.

This system of governance as well as the GitHub permissions model is subject to change as the community grows larger. Documents describing government bylaws, release, and contribution procedures are available in the GitHub repository.

## 3 SNIRF File Format

In this section, we present a brief overview of the specification and its features. The complete and authoritative specification can be found at https://github.com/fNIRS/snirf.

SNIRF is flexible to serve various NIRS applications, combining descriptions of raw data, processed data, auxiliary data, stimulus information, probe geometries, and experiment metadata. Because the file’s organizational groups are hierarchical, repeatable, and scalable, multiple measurements or experiments can be included in a single file, if desired.

### 3.1 HDF5

The SNIRF specifies an implementation of the HDF5 file format.^20^ HDF5 is a binary file format for storing data that is hierarchical and heterogeneous. It is suitable for encoding various numeric collections of data and probing geometry alongside human-readable collections of descriptors and metadata. The HDF5 file format supports online data and compression, facilitating efficient storage of large datasets and sparse signals. HDF5 has been in use by the scientific community since the early 2000s and is complete with mature and sustainable tools for reading and writing available in nearly all programming environments, including C/C++ and popular scripting platforms such as R, MATLAB, and Python.

### 3.2 Indexed Groups

HDF5 files are composed of datasets and groups. Datasets are units of data, such as strings and numeric values or collections of these, whereas groups serve as hierarchical parents to datasets and/or other groups.

**Fig. 1:**
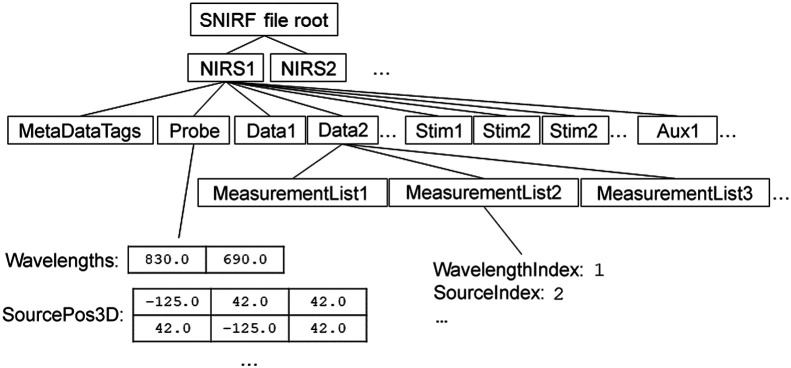
A typical SNIRF file hierarchy including only a few example fields and indexed groups. Any number of “nirs” indexed groups may exist at the file root. These may represent various experiments or subjects, such as each of the subjects in a dyadic recording. Each “nirs” group contains metadata and probe information as well as some number of stimulus descriptors, auxiliary signals, and fNIRS data. Each data group contains a matrix of fNIRS channels called “dataTimeSeries” in addition to the measurement list structure that describes these channels (pictured in Fig. 2).

The SNIRF specification introduces the notion of “indexed groups” as an extension of the HDF5 group: groups may form collections with groups of the same name, indexed starting at 1, i.e., stim1, stim2, and stim3. This feature allows for organizational units such as channels of data, experimental stimulus signals, or auxiliary signals to be repeated in the file indefinitely. Figure 1 shows a typical SNIRF file’s arrangement of indexed groups.

### 3.3 Measurement List

An fNIRS dataset necessarily includes complex relationships between time-varying signals and their descriptors. Without connections between a set of signals and the probe used to acquire it, data analysis and image reconstruction are impossible.

**Fig. 2:**
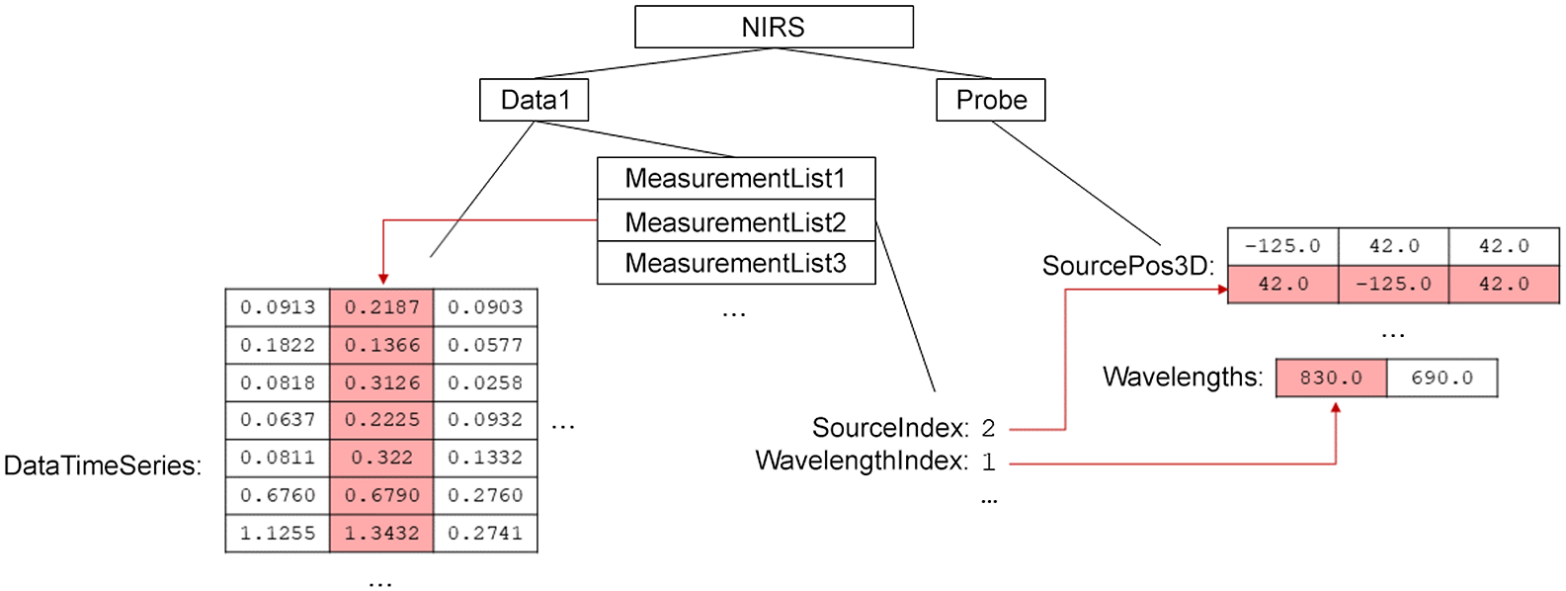
A typical measurement list relationship with a channel of data in “dataTimeSeries” and the corresponding probe data. The second element of the measurement list “measurementList2” corresponds to the second column of “dataTimeSeries”. This measurement list element contains integers “sourceIndex” and “wavelengthIndex,” which are indices into arrays that belong to the “probe” group: “sourcePos3D” and “wavelengths”. This indicates that the measured signal stored in the second channel of “dataTimeSeries” was originally produced at the optode with three-dimensional coordinate [42.0, −125.0, 42.0] by a source with a nominal wavelength of 830 nm. An analagous index for the coordinates of the channel’s detector is not shown. There are a variety of probe geometry formats supported by SNIRF.

The measurement list is an indexed group structure that links the data time series to its corresponding probe, thereby connecting it with probe features such as source channel position, detector channel position, and source wavelength. Because the capability of SNIRF encompasses processed data as well as time domain and frequency domain measurements, each measurement list element may include fields that describe the units and type of data in the channel, irrespective of the probe. Figure 2 depicts a measurement list element and its indices into the fNIRS signals and probe data.

### 3.4 Metadata Tags

To ensure that SNIRF files are as self-describing as possible, a flexible list of metadata tags exists for each set of data within the file (each “nirs” element). The user may add any data they wish to the metadata tags Group, but several entries are required by the SNIRF specification, including a subject identifier, the time of acquisition, and the units of the data.

### 3.5 Required Data

The minimum SNIRF file includes at least one series of raw data and a description of the probe used to acquire it, as well as several metadata tag entries. Additional content is optional, facilitating more detailed descriptions of fNIRS signals or the inclusion of additional data, such as accelerometer recordings or experimental stimuli. Signals in a single SNIRF file may have various acquisition frequencies and onsets.

## 4 Software

The SNIRF specification is an ambitious format that covers a wide variety of NIRS data types and allows for storing data from a range of experimental designs. This results in a complex file format with a variety of optional and interrelated data fields. To assist in the development of software, to remove implementation ambiguity, and to allow users to ensure their files are compliant with the SNIRF specification (and thus compatible with any SNIRF compliant software), a SNIRF file validator was developed as part of the Python package pysnirf2. The validator will assess files for compliance with the current version of the SNIRF specification, providing human-readable and machine-readable descriptions of any errors.

Code samples demonstrating the writing of SNIRF-compliant datasets in MATLAB and Python are available in the SNIRF repository at https://github.com/fNIRS/snirf, along with sample files and datasets. Additionally, the Homer3 software package provides a MATLAB interface for reading and writing SNIRF files.

## 5 Future Work

As an open-source format, SNIRF will expand in utility and robustness with its community of users and developers. Thus, we will continue to encourage stakeholders to get involved with the development and design of the specification. Ensuring that SNIRF is compatible with the latest fNIRS software and hardware is an ongoing effort. Although SNIRF was designed to support time domain measurements and diffuse correlation spectroscopy, these modalities are not as popular as continuous-wave approaches. We will encourage users of these emerging modalities to engage with the development of SNIRF to make this support more robust. So far, Kernel has successfully implemented time domain time SNIRF in a commercially available system.

Efforts to introduce SNIRF to neuroimaging standards ecosystems, such as the Brain Imaging Data Structure (BIDS),^21^ Neuroscience Without Borders,^22^ and NeuroJSON,^23^ are underway; these will enable the creation of standardized multimodal datasets including fNIRS, as well as the sharing of these datasets on online archival repositories such as OpenNeuro.^24^

## 6 Conclusion

SNIRF provides a versatile and widely supported standard for fNIRS data. The SNIRF specification was developed in collaboration with the fNIRS community, hardware vendors, and software developers. Currently, hardware vendors Artinis Medical Technologies, NIRx Medical Technologies, Kernel, Gowerlabs, OBELAB, and Cortivision allow their users to export data in SNIRF format. This allows users to easily import their data into any of the software platforms that can read SNIRF files; this list currently includes Homer3, MNE, FieldTrip, AnalyzIR, Satori, and BrainStorm.

The SNIRF specification will push forward the scientific community by providing a community-driven framework for hardware and software platforms and enabling efficient acquisition, processing, and sharing of data.

## Code, Data, and Materials Availability

The SNIRF specification and links to supporting documentation and software can be found at https://github.com/fNIRS/snirf. The SNIRF validator and Python interface can be found at https://doi.org/10.5281/zenodo.6784209. A sample SNIRF dataset that demonstrates the BIDS extension proposal for fNIRS can be found at https://doi.org/10.5281/zenodo.6575155.
